# miR-137 boosts the neuroprotective effect of endothelial progenitor cell-derived exosomes in oxyhemoglobin-treated SH-SY5Y cells partially via COX2/PGE2 pathway

**DOI:** 10.1186/s13287-020-01836-y

**Published:** 2020-10-26

**Authors:** Yuchen Li, Jinju Wang, Shuzhen Chen, Pei Wu, Shancai Xu, Chunlei Wang, Huaizhang Shi, Ji Bihl

**Affiliations:** 1 Department of Pharmacology & Toxicology, Boonshoft School of Medicine, Wright State University, Dayton, OH 45435 USA; 2 Department of Neurosurgery, The First Affiliated Hospital, Harbin Medical University, Harbin, 150000 Heilongjiang China; 3 Department of Biomedical Science, Joan C. Edwards School of Medicine, Marshall University, Huntingto, WV 25755 China

**Keywords:** miR-137, Oxyhemoglobin, Exosomes, COX2, Ferroptosis

## Abstract

**Background:**

We have previously verified the beneficial effects of exosomes from endothelial progenitor cells (EPC-EXs) in ischemic stroke. However, the effects of EPC-EXs in hemorrhagic stroke have not been investigated. Additionally, miR-137 is reported to regulate ferroptosis and to be involved in the neuroprotection against ischemic stroke. Hence, the present work explored the effects of miR-137-overexpressing EPC-EXs on apoptosis, mitochondrial dysfunction, and ferroptosis in oxyhemoglobin (oxyHb)-injured SH-SY5Y cells.

**Methods:**

The lentiviral miR-137 was transfected into EPCs and then the EPC-EXs were collected. RT-PCR was used to detect the miR-137 level in EPCs, EXs, and neurons. The uptake mechanisms of EPC-EXs in SH-SY5Y cells were explored by the co-incubation of Dynasore, Pitstop 2, Ly294002, and Genistein. After the transfection of different types of EPC-EXs, flow cytometry and expression of cytochrome c and cleaved caspase-3 were used to detect the apoptosis of oxyHb-injured neurons. Neuronal mitochondrial function was assessed by reactive oxygen species (ROS) level, mitochondrial membrane potential (MMP) depolarization, and cellular ATP content. Cell ferroptosis was measured by lipid peroxidation, iron overload, degradation of glutathione, and glutathione peroxidase 4. Additionally, recombinational PGE2 was used to detect if activation of COX2/PGE2 pathway could reverse the protection of miR-137 overexpression.

**Results:**

The present work showed (1) EPC-EXs could be taken in by SH-SY5Y cells via caveolin-/clathrin-mediated pathways and macropinocytosis; (2) miR-137 was decreased in neurons after oxyHb treatment, and EXs^miR-137^ could restore the miR-137 levels; (3) EXs^miR-137^ worked better than EXs in reducing the number of apoptotic neurons and pro-apoptotic protein expression after oxyHb treatment; (4) EXs^miR-137^ are more effective in improving the cellular MMP, ROS, and ATP level; (5) EXs^miR-137^, but not EXs, protected oxyHb-treated SH-SY5Y cells against lipid peroxidation, iron overload, degradation of glutathione, and glutathione peroxidase 4; and (6) EXs^miR-137^ suppressed the expression of the COX2/PGE2 pathway, and activation of the pathway could partially reverse the neuroprotective effects of EXs^miR-137^.

**Conclusion:**

miR-137 overexpression boosts the neuroprotective effects of EPC-EXs against apoptosis and mitochondrial dysfunction in oxyHb-treated SH-SY5Y cells. Furthermore, EXs^miR-137^ rather than EXs can restore the decrease in miR-137 levels and inhibit ferroptosis, and the protection mechanism might involve the miR-137-COX2/PGE2 signaling pathway.

## Introduction

Stroke is the second leading cause of death and the main cause of adult disability worldwide [1]. According to its pathological mechanisms, stroke can be divided into two types: ischemic stroke and hemorrhagic stroke. Hemorrhagic stroke poses a deadlier threat and a more serious burden to public health [2]. Though interventions for and clinical management of stroke have improved, the poor prognosis caused by secondary brain injury is not reversed in patients with hemorrhagic stroke [3, 4]. However, the exact mechanisms underlying secondary brain injury and effective treatments for it have not been illuminated. In studies of hemorrhagic stroke, oxyhemoglobin (oxyHb), a major component of blood, is widely used in in vitro models of intracerebral hemorrhage (ICH) and subarachnoid hemorrhage (SAH) [5, 6].

Exosomes (EXs) are released by nearly all types of cells and appear as vesicles surrounded by lipid bilayer membranes in all body fluids [7]. EXs are approximately 30–150 nm in diameter and contain multifarious molecules including proteins, RNA, DNA, and other molecules [8]. EXs are thought to be important transfer vectors for intercellular communication and can regulate their target areas [9]. EXs are reported to be involved in the pathological and physiological mechanisms of neurological diseases, such as cerebral trauma, tumor, neuroinflammation, and neurodegeneration [10–12]. Specifically, EXs are verified to have various roles in brain repair and may serve as circulating biomarkers in stroke [13]. In a study of ICH, EXs from mesenchymal stem cells (MSCs) were found to improve axonal and white matter injury in experimental animal models [14]. Our group previously reported that endothelial progenitor cell (EPC)-released EXs could protect endothelial cells (ECs) against hypoxia/reoxygenation (H/R) injury, which is partially mediated by miR-210 [15].

MicroRNAs (miRNAs), a large class of non-coding RNAs, regulate multiple biological processes in a wide range of organisms, including animals, plants, and viruses [16]. miR-137 is a brain-enriched miRNA, and its expression is high in the cortex and hippocampus and low in the cerebellum and brain stem; it can regulate neuronal development, differentiation, and maturation via a large number of downstream target genes in various pathways [17]. miR-137 also plays a key role in the pathology of neurological disorders. In spinal cord injury, miR-137 was reported to attenuate inflammation and oxidative stress in mice by modulating NEUROD4 [18]. Liu et al. found that upregulation of miR-137 could promote EPC proliferation and angiogenesis in mice after cerebral ischemic stroke through the Notch pathway [19]. In cancer, miR-137 was found to suppress gastric carcinogenesis by targeting cyclooxygenase 2 (COX2) [20]. Previous studies also found that a combination of EXs and miRNAs could provide more benefit to the nervous system in pathological conditions. The EXs derived from miR-133b-overexpressing MSCs have been reported to improve neural plasticity and functional recovery after ischemic stroke [21]. Our previous work found that transfection of miR-210 into EPCs-EXs could enhance the beneficial effects on mitochondrial function in H/R-injured human ECs [15]. However, few studies have focused on the combination of EXs and miRNAs, especially miR-137, in hemorrhagic stroke.

In the present study, we used oxyHb-treated SH-SY5Y cells to imitate hemorrhagic stroke models in vitro. We transfected miR-137 to EXs derived from EPCs, and the miR-137-overexpressing EXs were used to explore their neuroprotective effects against apoptosis, mitochondrial dysfunction, and ferroptosis in oxyHb-treated cells. In our mechanism study, we determined whether the COX2/prostaglandin E_2_ (PGE2) pathway acted as the downstream pathway of miR-137-overexpressing EXs.

## Materials and methods

### Cell culture

Human neuroblastoma cell line SH-SY5Y cells (ATCC, MD, USA) were cultured in complete medium containing Ham’s F-12 K Nutrient Mixture and Eagle’s Minimum Essential Medium (Corning Cellgro, VA, USA) with 10% fetal bovine serum (HyClone, PA, USA), 100 IU/ml penicillin, and 100 mg/ml streptomycin in an atmosphere of 5% CO_2_ at 37 °C. The medium was changed every other day, the cells were passaged when the density reached around 75%. The 12–16 passages of the cells were used in the present study.

Human endothelial progenitor cells (Celprogen, Torrance, CA) were cultured in complete growth medium (Celprogen, Torrance, CA) under standard cell culture conditions (5% CO_2_, 37 °C) according to the manufacturer’s protocol. The medium was changed every other day, and the cells were passaged when the density reached around 80%. The cells underwent 8–11 passages in the present study.

### Oxyhemoglobin-induced neuron injury model

For the in vitro neuron injury model, oxyHb was used to mimic the injury that occurs to neurons in the brain after hemorrhagic stroke [5, 6]. In brief, the SH-SY5Y cells were seeded in culture plates and cultured with complete medium (5% CO_2_, 37 °C) for 24 h. Then, the medium was removed and the cells were exposed to 10 μM oxyHb (Sigma-Aldrich, MO, USA) in complete medium for 24 h. The dose and time point of oxyHb were selected according to our previous study [4]. Cells were then used for the following experiments.

### miR-137 transfection

**Fig. 1:**
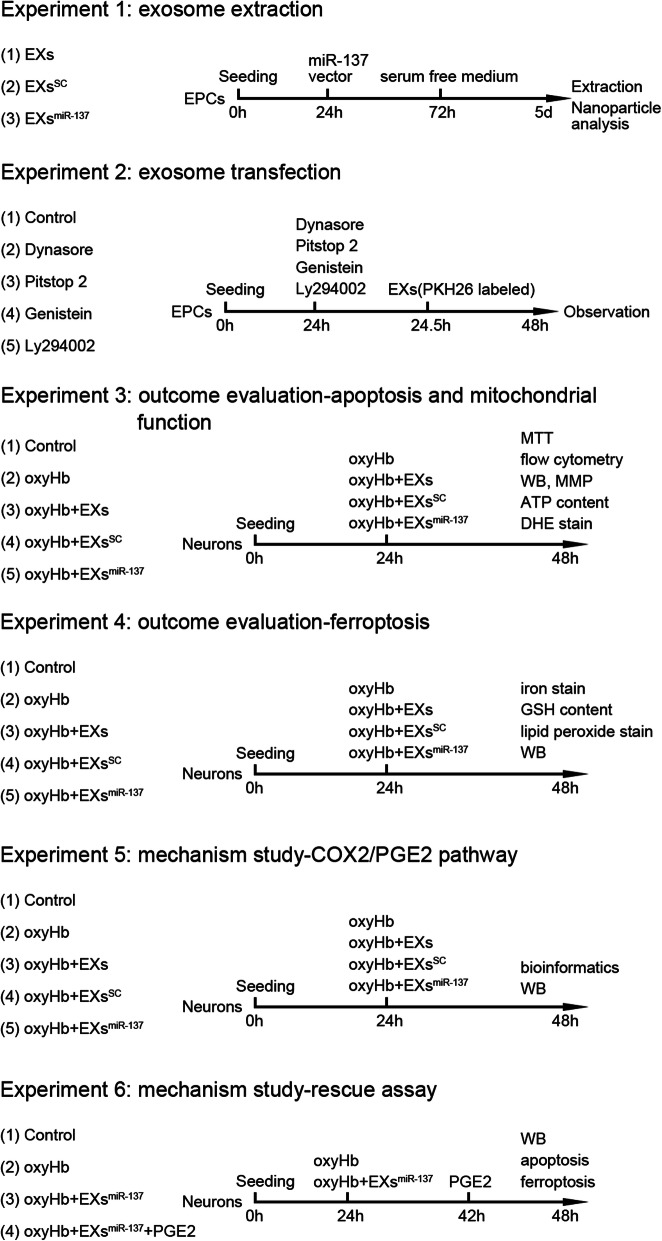
Experiment protocols. oxyHb, oxyhemoglobin; EPC, endothelial progenitor cell; EXs, exosomes; MTT, 3-(4,5-dimethylthiazol-2-yl)-2,5-diphenyltetrazolium bromide; DHE, dihydroethidium; MMP, mitochondrial membrane potential; GSH, glutathione; WB, western blot

To overexpress miR-137, the EPCs were cultured to 60–70% confluence, and then ShMIMIC Lentiviral microRNA (hsa-mir-137) and SMARTvector Empty Vector (1:125 diluted in complete growth medium, Dharmacon, CO, USA) were transfected into the EPCs for 48 h. Along with the control EPCs that were cultured in complete growth culture medium, three types of EPCs were used to produce corresponding EXs (Fig. 1). We determined the transfection rate in EPCs, and whether the Lentiviral miRNAs (hsa-mir-137) with green fluorescence were also transfected into the SH-SY5Y cells via EXs. After miR-137/EXs^miR-137^ transfection, the EPCs/SH-SY5Y cells were fixed with 4% paraformaldehyde (PFA) and stained with 4′,6-diamidino-2-phenylindole (DAPI) dye. Fluorescence was observed using the EVOS cell imaging system (Thermo Fisher Scientific, MA, USA) and analyzed with Image J software (Image J 1.4, NIH, USA).

### Exosome extraction

The EXs were collected from the medium of EPCs according to our previously reported method [15]. Briefly, the three types of EPCs were cultured to 70–80% confluence and then incubated with serum-free culture medium (Celprogen, Torrance, CA) for 48 h. Then, the medium was collected and centrifuged at 2000*g* for 20 min to remove dead cells. The supernatants were centrifuged at 20,000*g* for 70 min and ultracentrifuged at 170,000*g* for 90 min to pellet EXs. The pelleted EXs, including EPC-EXs, EPC-EXs^SC^, and EPC-EXs^miR-137^, were resuspended with phosphate-buffered saline (PBS) and then aliquoted for nanoparticle tracking analysis (NTA) and the co-incubation study. PBS was filtered through a 20-nm filter (Whatman, Pittsburgh, PA).

### Co-incubation study

The SH-SY5Y cells were divided into different co-incubation groups as shown in Fig. 1. In the control group, cells were only incubated with complete medium. In the oxyHb group, cells were incubated with 10 μM oxyHb in complete medium for 24 h. In the oxyHb+EXs, oxyHb+EXs^SC^, and oxyHb+EXs^miR-137^ groups, cells were co-incubated with EPC-EXs, EPC-EXs^SC^, and EPC-EXs^miR-137^, respectively for 24 h. Each type of EXs (1 × 10^9^) was diluted in 10 μM oxyHb in complete medium for co-incubation. For the mechanism study, recombinational PGE2 (Sigma-Aldrich, MO, USA) was used to activate the COX2/PGE2 pathway. For the oxyHb+EXs^miR-137^ + PGE2 group, PGE2 (10 mM, diluted in distilled water) was added to the complete medium at a final concentration of 100 ng/ml and then cells were co-incubated with PGE2 (100 ng/ml), oxyHb (10 μM), and EXs^miR-137^ (1 × 10^9^) in complete medium for 24 h.

To determine whether EPC-EXs, EPC-EXs^SC^, and EPC-EXs^miR-137^ were successfully transfected into SH-SY5Y cells, the three types of EXs were labeled with a red fluorescence dye PKH 26 (Sigma-Aldrich, MO, USA) according to the manufacturer’s protocol and were then co-cultured with SH-SY5Y cells for 24 h. After that, the cells were washed with PBS once and fixed with 4% PFA for 10 min. After washing with PBS twice, the cells were stained with DAPI solution for 2 min and washed with PBS twice again. Fluorescence was observed using the EVOS cell imaging system. The fluorescence intensity of EXs in cells was analyzed using Image J software (Image J 1.4, NIH, USA).

To further explore the uptake mechanisms of EXs in SH-SY5Y cells, four different inhibitors (Sigma-Aldrich, MO, USA) of major EX uptake pathways were added prior to EPC-EXs co-incubation. Dynasore (80 μM, dynamin inhibitor), Genistein (200 μM, caveolin-mediated pathway inhibitor), Pitstop 2 (10 μM, clathrin-dependent pathway inhibitor), and Ly294002 (5 μM, macropinocytosis inhibitor) were diluted with complete medium and co-incubated with the cells for 25 min, and the cells were then co-incubated with EPC-EXs labeled with PKH 26 in complete medium for 24 h. Fluorescent images were taken using the EVOS cell imaging system and analyzed with Image J software (Image J 1.4, NIH, USA).

### Nanoparticle tracking analysis

The NanoSight NS300 (Malvern Instruments, Malvern, UK) was used to analyze the size and concentration of EXs. The collected EXs in each group were first resuspended with 100 μl PBS and then separated into 10 μl aliquots of the suspension and diluted 1 in 100 with PBS (990 μl). The PBS was filtered through a 20-nm filter. Subsequently, the samples were analyzed on the NanoSight NS300. Three 30-s videos were taken with a frame rate of 30 frames per second. The results were analyzed using the NTA 3.0 software (Malvern Instruments, Malvern, UK) on a frame-by-frame basis.

### Quantitative real-time polymerase chain reaction (RT-PCR) analysis

Total RNA was extracted from EPCs, EPC-EXs, and SH-SY5Y cells in each group using Trizol (Thermo Fisher Scientific, MA, USA). To quantify the miR-137 levels, reverse transcription (RT) reactions were performed using the PrimeScriptTM RT reagent kit (TaKaRa, Japan), and PCR reactions were performed using SYBR Premix EX TaqTM II kit (TaKaRa, Japan). The RT primer for miR-137 was as follows: 5′-GTCGTATCCAGTGCAGGGTCCGAGGTATTCGCACTGGATACGACATTATC-3′, the forward primer was 5′-GCGCGCTTATTGCTTAAGAATAC-3′, and the reverse primer was 5′- GTGCAGGGTCCGAGGT-3′. U6 was used as an endogenous control; the forward primer for U6 was 5′-CTCGCTTCGGCAGCACA-3′, and the reverse primer was 5′-AACGCTTCACGAATTTGCGT-3′. Relative expression of miR-137 was normalized to U6, and the results were calculated using the 2^-ΔΔCT^ method.

### Cell viability

The cell viability in every group was measured with an MTT (Sigma-Aldrich, MO, USA) assay. The cells were seeded into 96-well plates at a concentration of 2 × 10^3^ cells/well containing 100 μl of complete medium. After co-incubation, MTT solution (20 μl, 5 mg/ml) was added to each well and incubated at 37 °C for 4 h. Then 150 μl of dimethyl sulfoxide (DMSO) was added to each well and incubated at 37 °C for 20 min. The results were measured on a microplate reader (BioTek, USA). The optical density (OD) was read at 490 nm according to the manufacturer’s protocol, and each group had triplicate wells.

### Apoptosis assay

After 24 h of co-culture, the SH-SY5Y cells were detached for the apoptosis assay using the PI/Annexin V stain (BD Biosciences, CA, USA) method, according to the manufacturer’s protocol. The cells in each group were stained with PI dye (10 μl) and Annexin V dye (5 μl) for 15 min at room temperature. Then, the results were analyzed with Accur C6 Plus flow cytometry (BD Biosciences, CA, USA). The apoptotic index was calculated as follows: Annexin V+/PI− cells/total cells × 100% [22].

### Dihydroethidium staining

The intracellular ROS production was determined using dihydroethidium (DHE) (Sigma-Aldrich, MO, USA) staining as previously reported [23]. The result was analyzed using flow cytometry. Data are expressed as fold of control in fluorescent intensity.

### Mitochondrial membrane potential assay

The mitochondrial membrane potential (MMP) of SH-SY5Y cells in each group was measured using the lipophilic cationic dye JC-1 (1:1000, Invitrogen, Carlsbad, CA, USA) according to our previously published method [15]. In brief, after co-incubation, cells were washed with PBS and incubated with the JC-1 staining probe (2 μM in complete medium) for 30 min at 37 °C. The cells were then washed with PBS and observed under a fluorescence microscope (EVOS; Thermo Fisher Scientific, MA, USA). The level of cellular fluorescence intensity was analyzed using Image J (Image J 1.4, NIH, USA). The relative MMP was calculated as the ratio of J-aggregate to monomer (590/520 nm). The results are expressed as fold of control cells. The micrographs were taken with the same gain/intensity with the same threshold. Researchers blinded to the grouping information performed the analysis.

### ATP content

The ATP content in each group was detected with the ATP Assay Kit (Abcam, Cambridge, UK). Briefly, cells were harvested after co-incubation and washed with cold PBS and then resuspended in 100 μl of ATP Assay Buffer and centrifuged at 13,000*g* for 5 min. The supernatants were transferred to new tubes, and the ATP reaction mixtures were prepared according to the manufacturer’s protocol. The optional OD of 570 nm was used on a microplate reader (BioTek, USA). The ATP content (nmol) was calculated against the standard curve.

### Glutathione content

Glutathione (GSH) content in the cells was measured with the GSH-Glo™ Glutathione Assay (Madison, WI, USA). Cells were seeded into a 96-well plate and different groups were tagged. After co-incubation, the culture medium in the wells was carefully removed; then 100 μl of prepared 1X GSH-Glo Reagent was added to each well and incubated at room temperature for 30 min. After that, 100 μl of reconstituted Luciferin Detection Reagent was added to each well of a 96-well plate and incubated at room temperature for 15 min. Luminescence was measured using a microplate reader (BioTex, VT, USA). The results were calculated by subtracting the luminescence of the negative control reactions from that of GSH-containing reactions.

### Iron stain assay

Iron deposition in SH-SY5Y cells of each group was detected with an Iron Stain Kit (Sigma-Aldrich, MO, USA). According to the manufacturer’s protocol, the fixed cells were stained with iron staining solution and then pararosanline solution. Micrographs were taken using an Olympus DP74 microscope (Olympus Co., Tokyo, Japan). The quantitative analysis of the relative iron-stained area was performed using Image J software (Image J 1.4, NIH, USA).

### Lipid peroxide fluorescence staining

Accumulation of lipid peroxide in cells was determined using the Image-iT Lipid Peroxidation Kit (Molecular Probes, OR, USA). Briefly, cells were seeded in a 6-well plate at a density of 5 × 10^4^ and incubated for 24 h at 37 °C. After co-incubation, the staining solution (10 μM) was added to the cells and incubated for 30 min at 37 °C. Then, the cells were fixed with 4% PFA solution and counterstained with DAPI (1 μm/ml, Thermo Fisher Scientific, MA, USA). Micrographs were taken using the EVOS cell imaging system (Thermo Fisher Scientific, MA, USA). The fluorescence ratio of Texas Red/FITC revealed lipid peroxidation and was analyzed with Image J software (Image J 1.4, NIH, USA).

### Western blot

The cells were homogenized in ice-cold lysis buffer (Thermo Fisher Scientific, MA, USA) for protein extraction. The total protein concentration was determined using the BCA protein assay (Bio-Rad, CA, USA). After electrophoresis, the protein samples were transferred to a 0.45-μm polyvinylidene fluoride (PVDF) membrane. The membrane was blocked in 5% non-fat milk for 1 h at room temperature and incubated with primary antibodies at 4 °C overnight. The primary antibodies included glutathione peroxidase 4 (GPx4, 1:500, Sigma-Aldrich, MO, USA), cleaved caspase-3 (1:1000, Santa Cruz, TX, USA), cytochrome C (Cyt-C, 1:1000, Abcam, Cambridge, UK), COX2 (1:500, Abcam, Cambridge, UK), PGE2 (1:500, Bioss, MA, USA), glyceraldehyde 3-phosphate dehydrogenase (GAPDH; 1:200, Santa Cruz, TX, USA), and β-actin (1:4000, Sigma-Aldrich, MO, USA). The membrane was then incubated with anti-rabbit or anti-mouse horseradish peroxidase (HRP)-conjugated secondary antibodies for 1 h at room temperature, and protein bands were visualized with the Odyssey Infrared Imaging System (Licor Biosciences, Lincoln, NE, USA). Relative intensity was analyzed with Image J software (Image J 1.4, NIH, USA), and β-actin or GAPDH were used as endogenous controls.

### Statistical analysis

Data are presented as mean ± standard deviation (SD). We compared every two groups with a two-tailed Student’s *t* test followed by Welch’s correction. The one-way analysis of variance (ANOVA) was used for comparisons among multiple groups. Bonferroni or Dunn’s post hoc analyses were used to determine where differences occurred. All analyses were carried out using GraphPad Prism 5.0 Software (GraphPad Software, La Jolla, CA, USA). The criterion for statistical significance was *P* < 0.05.

## Results

### Characteristics of EPC-EXs and uptake pathways in SH-SY5Y cells

**Fig. 2:**
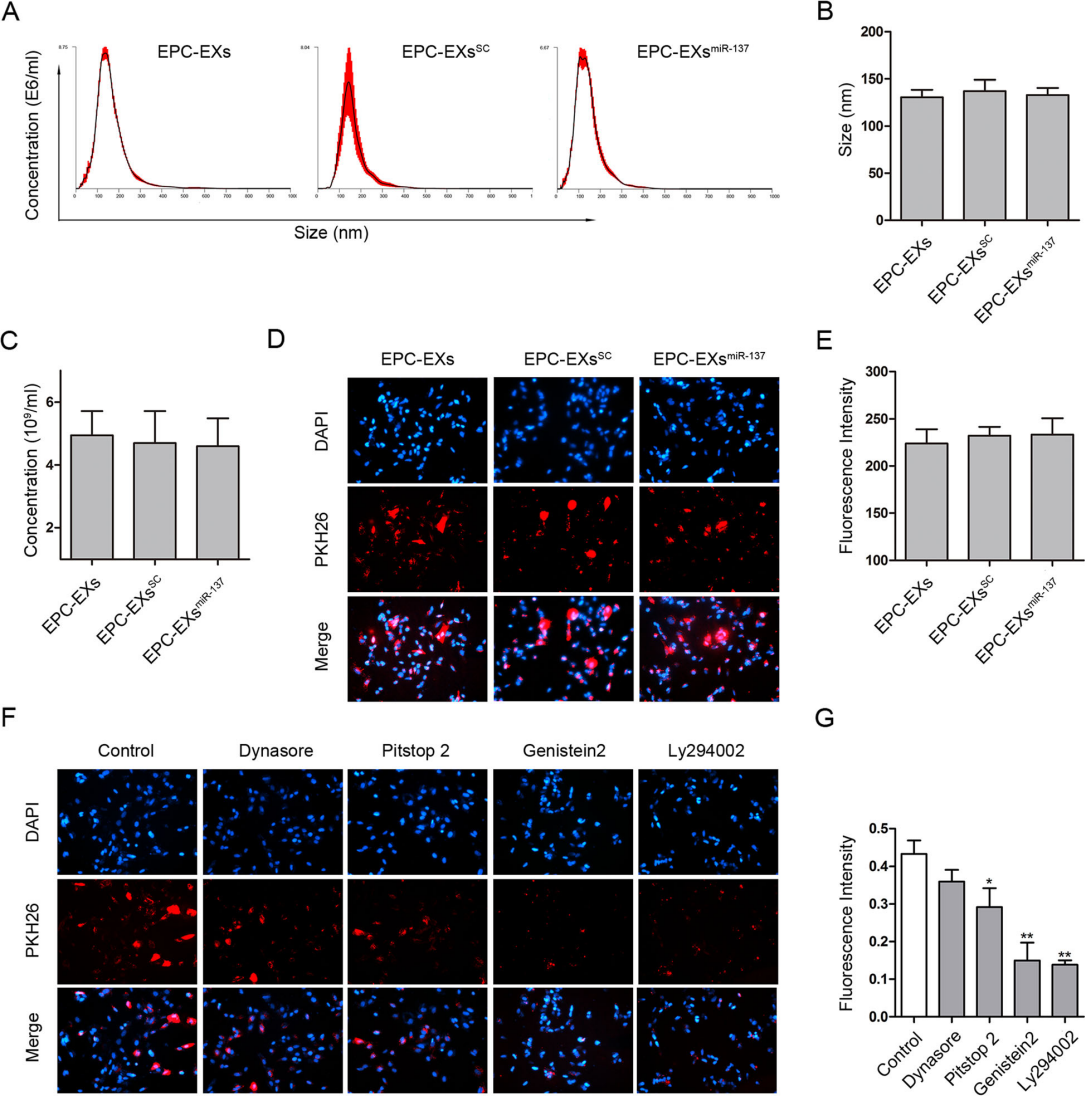
Characterization of EPC-EXs and transfection of EPC-EXs into SH-SY5Y cells. **a** Representative results from the nanoparticle analysis of EPC-EXs. **b** The size of EPC-EXs, EPC-EXs^SC^, and EPC-EXs^miR-137^ were comparable, *n* = 6. **c** There were no differences on the concentration of EPC-EXs, EPC-EXs^SC^, and EPC-EXs^miR-137^, *n* = 6. **d** Representative micrographs of PKH26-labeled EPC-EXs in SH-SY5Y cells of different groups. **e** The amount of EPC-EXs, EPC-EXs^SC^, and EPC-EXs^miR-137^ took up by the neurons was comparable, *n* = 6. **f** Representative micrographs of PKH26-labeled EPC-EXs in SH-SY5Y cells after the administration of inhibitors involved in the major EXs uptake pathways. **g** Pitstop 2, Gebistein, and Ly294002 decreased the fluorescence intensity of EPC-EXs in SH-SY5Y cells, *n* = 6. *P < 0.05 and **P < 0.01 vs. control group

The EXs in each group were extracted by ultracentrifugation and analyzed using the nanoparticle tracking system. A representative result is shown in Fig. 2a. The data show that the average diameter of the EXs in the three different groups was less than 150 nm, and there was no significant difference in the size or concentration of EXs between the EPC-EXs, EPC-EXs^SC^, and EPC-EXs^miR-137^ groups (*p* > 0.05, Fig. 2b, c). The PKH 26-labeled EXs were found to be successfully transfected into SH-SY5Y cells (Fig. 2d). The relative fluorescence intensity showed that the amounts of the three types of EXs transfected into the cells were comparable (*p* > 0.05, Fig. 2e). Four inhibitors of the major EXs uptake pathways were used to explore the uptake mechanisms of EXs in neurons. The relative fluorescence intensity of EXs was partially decreased in the Pitstop 2 group (*p* < 0.05 vs. control), significantly decreased in both the Gebistein and Ly294002 groups (*p* < 0.01 vs. control), while there was no significant difference between the Dynasore and control groups (*p* > 0.05, Fig. 2f, g). These results indicate that EPC-EXs, EPC-EXs^SC^, and EPC-EXs^miR-137^ can be successfully and equally transfected into SH-SY5Y cells, and EXs might be taken up via caveolin-dependent and clathrin-mediated pathways and by macropinocytosis.

### EPC-EXs^miR-137^ increased miR-137 levels in oxyHb-treated SH-SY5Y cells

**Fig. 3:**
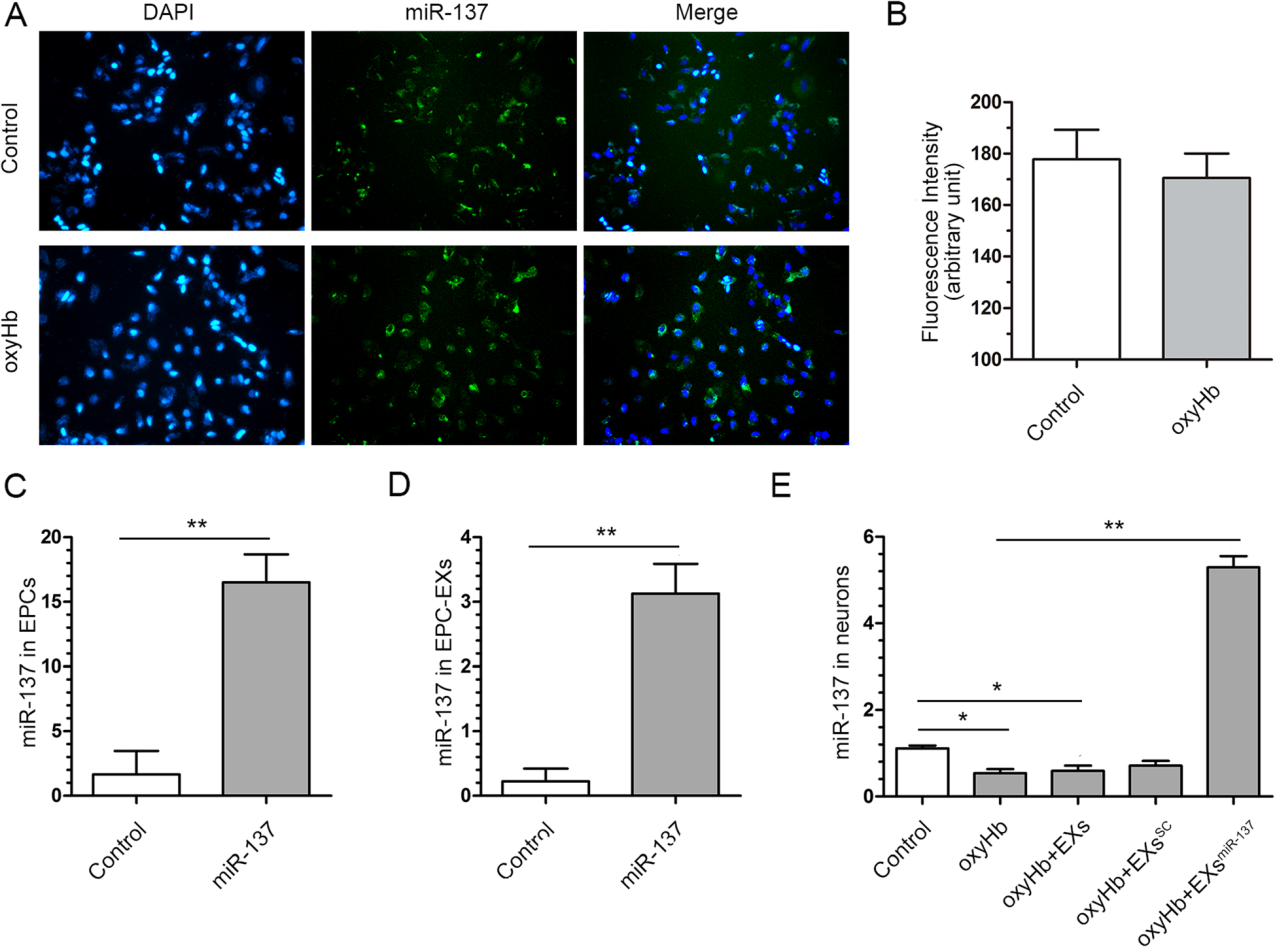
miR-137 level in oxyHb-treated SH-SY5Y cells was decreased and improved after EPC-EXs^miR-137^ transfection. **a** Representative micrographs showed miR-137 could be transfected into SH-SY5Y cells via EPC-EXs. **b** Fluorescence intensity of miR-137 was comparable between the control and oxyHb groups after the EPC-EXs^miR-137^ transfection, *n* = 6. **c** Lentiviral miR-137 transfection increased the miR-137 in EPCs, *n* = 6. **d** Lentiviral miR-137 transfection increased the miR-137 in EPC-EXs, *n* = 6. **e** EPC-EXs^miR-137^ increased the deteriorative miR-137 level in oxyHb-treated SH-SY5Y cells, *n* = 6. *P < 0.01 and **P < 0.01

As shown in Fig. 3a, miR-137 fluorescence can be observed in both the control and oxyHb-treated SH-SY5Y cells after EXs^mIR-137^ transfection. The relative fluorescence intensity of miR-137 in the SH-SY5Y cells treated with and without oxyHb was comparable (*p* > 0.05, Fig. 3b). Moreover, after transfection of miR-137, the miR-137 level in both EPCs and extracted EXs was significantly increased (*p* < 0.01 vs. control, Fig. 3c, d). RT-PCR was used to quantify the expression of miR-137 in the neurons of each group. miR-137 was downregulated after oxyHb treatment (*p* < 0.05 vs. control). EPC-EXs^miR-137^ abrogated the reduction in miR-137 levels (*p* < 0.05 vs. oxyHb), while EXs and EXs^SC^ did not change the expression of miR-137 in oxyHb-treated cells (*p* > 0.05 vs. oxyHb, Fig. 3e). These results indicate that miR-137 can be effectively transfected into SH-SY5Y cells via EXs, and oxyHb treatment did not influence the transfection rate; EXs^miR-137^ restore the reduced miR-137 levels in oxyHb-treated cells.

### EPC-EXs^miR-137^ decreased the neuronal apoptosis induced by oxyHb treatment

**Fig. 4:**
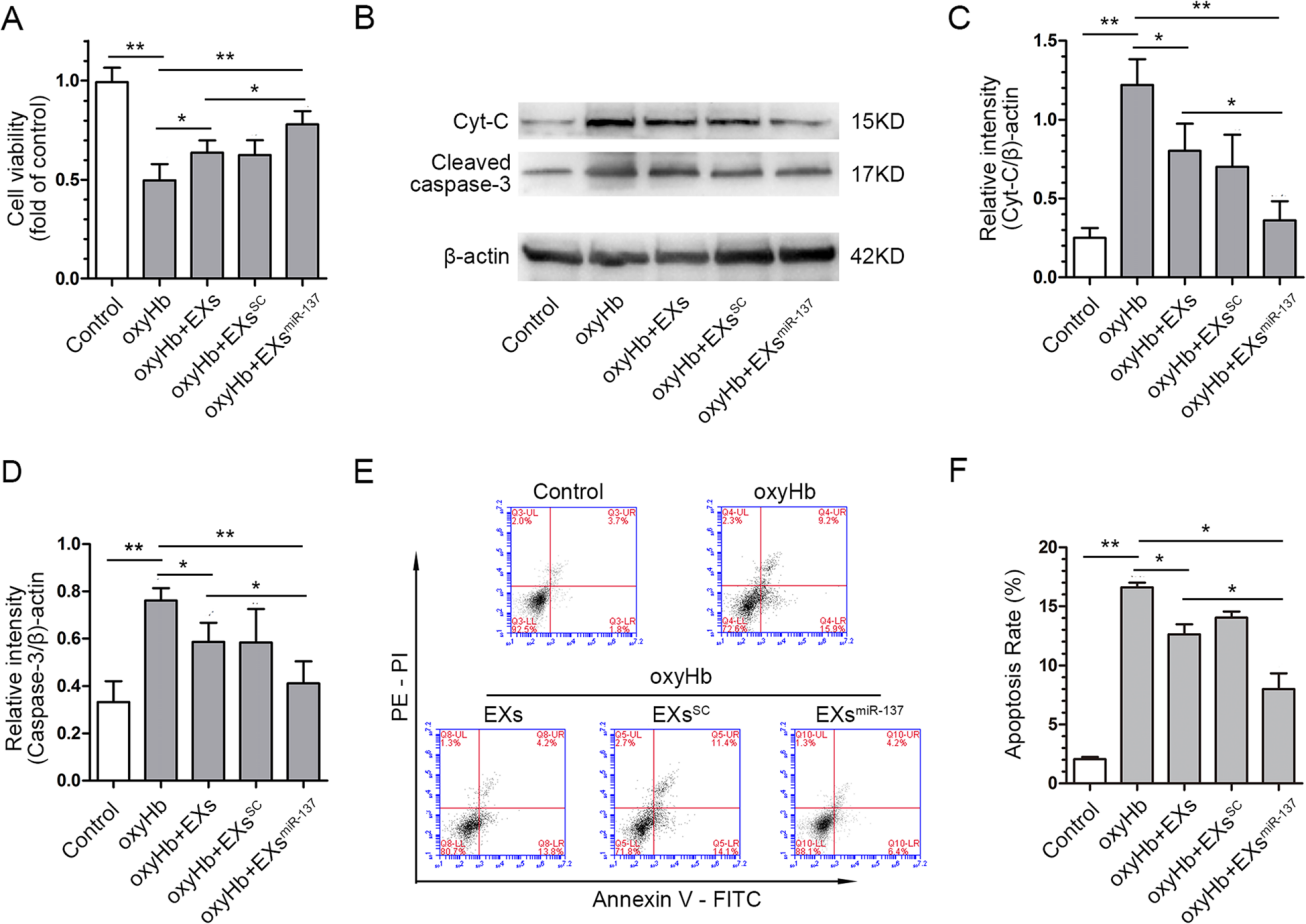
EPC-EXs^miR-137^ alleviated the oxyHb-induced apoptosis in SH-SY5Y cells. **a** Quantitative analysis of cell viability was detected by the MTT assay in each group, *n* = 6. **b** Representative bands of Cyt-C, cleaved caspase-3, and β-actin proteins in western blot revealed the different expression in each group; the densities of the protein bands were normalized to β-actin. **c** Quantification of relative expression of Cyt-C in each group, *n* = 4. **d** Quantification of relative expression of cleaved caspase-3 in each group, *n* = 4. **e** Representative Annexin V/PE staining result measured by flow cytometry. **f** Summarized data of the apoptosis rate in each group, *n* = 4. *P < 0.01 and **P < 0.01

The MTT assay revealed that cell viability was reduced in the oxyHb group (*p* < 0.01 vs. control) and improved in the oxyHb+EXs (*p* < 0.05 vs. oxyHb) and oxyHb+EXs^SC^ (*p* < 0.05 vs. oxyHb); the improvement is more obvious in oxyHb+EXs^miR-137^ groups (*p* < 0.01 vs. oxyHb, *p* < 0.05 vs. oxyHb+EXs, Fig. 4a). The expression of active caspase-3 and Cyt-C was used to detect apoptosis in SH-SY5Y cells. As shown in Fig. 4b, c, and d, both pro-apoptotic proteins were upregulated in oxyHb-treated cells (*p* < 0.01 vs. control), and while administration of EXs and EXs^SC^ could reduce their expression (*p* < 0.05 vs. oxyHb), EXs^miR-137^ were more effective than EXs and EXs^SC^ (*p* < 0.05). In addition, the flow cytometry results show that the apoptotic neurons were increased in the oxyHb group (*p* < 0.01 vs. control), while following the administration of EXs (*p* < 0.05 vs. oxyHb) and EXs^miR-137^ (*p* < 0.05 vs. oxyHb), the number of apoptotic neurons was decreased, and EXs^miR-137^ were more effective than EXs at inhibiting apoptosis (*p* < 0.05, Fig. 4e, f). These results indicate that miR-137 plays a role in the anti-apoptotic effect of EPC-EXs in oxyHb-treated SH-SY5Y cells.

### EPC-EXs^miR-137^ improved the mitochondrial dysfunction in oxyHb-treated SH-SY5Y cells

**Fig. 5:**
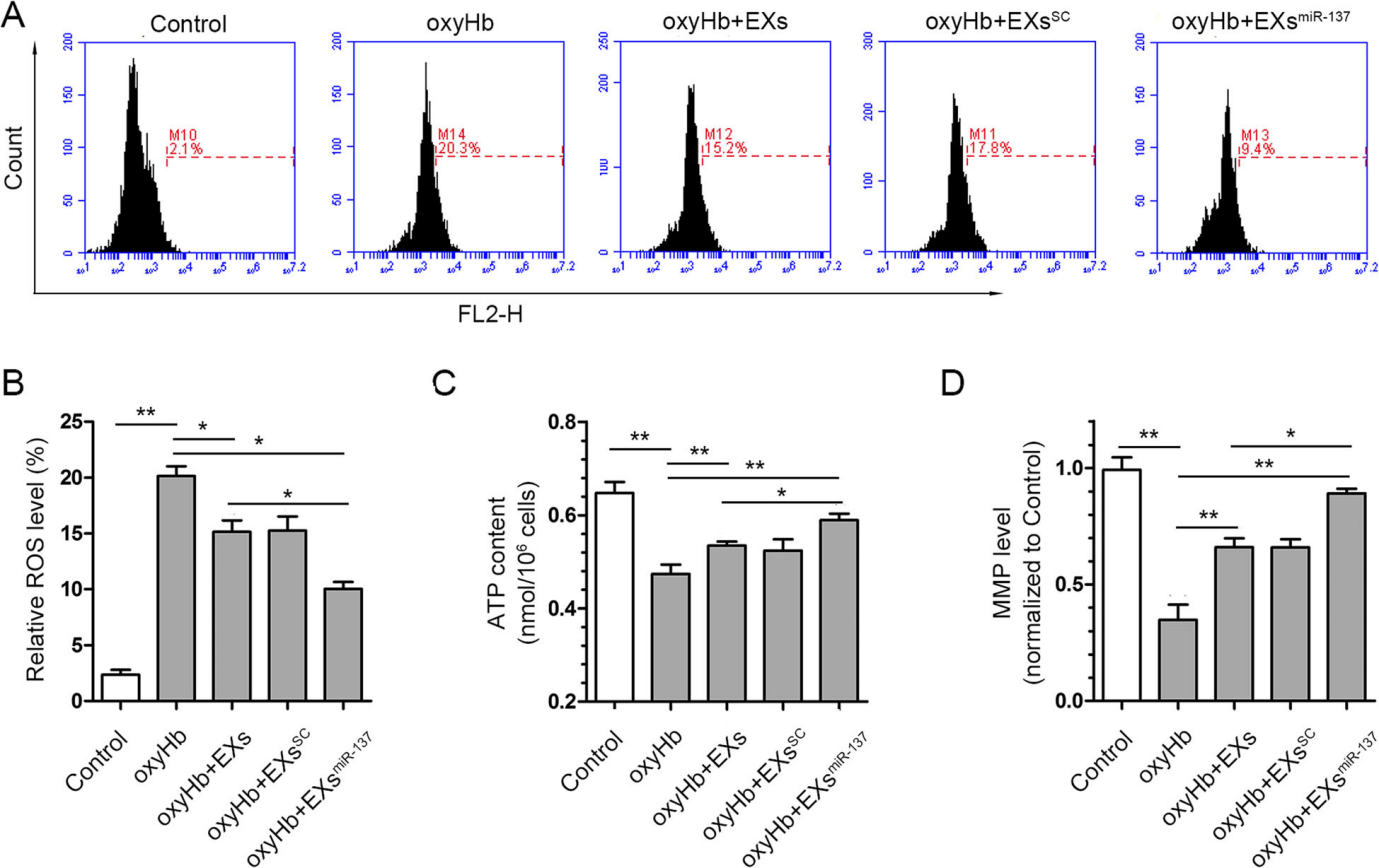
EPC-EXs^miR-137^ alleviated the oxyHb-induced mitochondrial dysfunction in SH-SY5Y cells. **a** Representative DHE staining result measured by flow cytometry. **b** Summarized data of the relative ROS level in each group, *n* = 4. **c** Quantification of ATP content in the neurons of each group, *n* = 6. **d** Quantification of MMP level in the neurons of each group, *n* = 6. *P < 0.01 and **P < 0.01

The ROS level measured by DHE staining in SH-SY5Y cells was significantly increased after oxyHb treatment (*p* < 0.01 vs. control). This was partially reversed in the oxyHb+EXs (*p* < 0.05 vs. oxyHb) and oxyHb+EXs^SC^ (*p* < 0.05 vs. oxyHb) groups, and further decreased in the oxyHb+EXs^miR-137^ group (*p* < 0.05 vs. oxyHb, *p* < 0.05 vs. oxyHb+EXs, Fig. 5a, b). The ATP content was also improved in the oxyHb+EXs (*p* < 0.01 vs. oxyHb) and oxyHb+EXs^SC^ (*p* < 0.05 vs. oxyHb) groups, and further increased in the oxyHb+EXs^miR-137^ group (*p* < 0.01 vs. oxyHb, *p* < 0.05 vs. oxyHb+EXs, Fig. 5c). EXs and EXs^SC^ could also restore the MMP level in oxyHb-treated cells (*p* < 0.01 vs. oxyHb), while EXs^miR-137^ were more effective at restoring MMP levels (*p* < 0.01 vs. oxyHb, *p* < 0.05 vs. oxyHb+EXs, Fig. 5d). These results indicate that miR-137 plays a role in the benefical effects of EPC-EXs on mitochondrial dysfunction in oxyHb-treated SH-SY5Y cells.

### EPC-EXs^miR-137^ alleviated the neuronal ferroptosis induced by oxyHb treatment

**Fig. 6:**
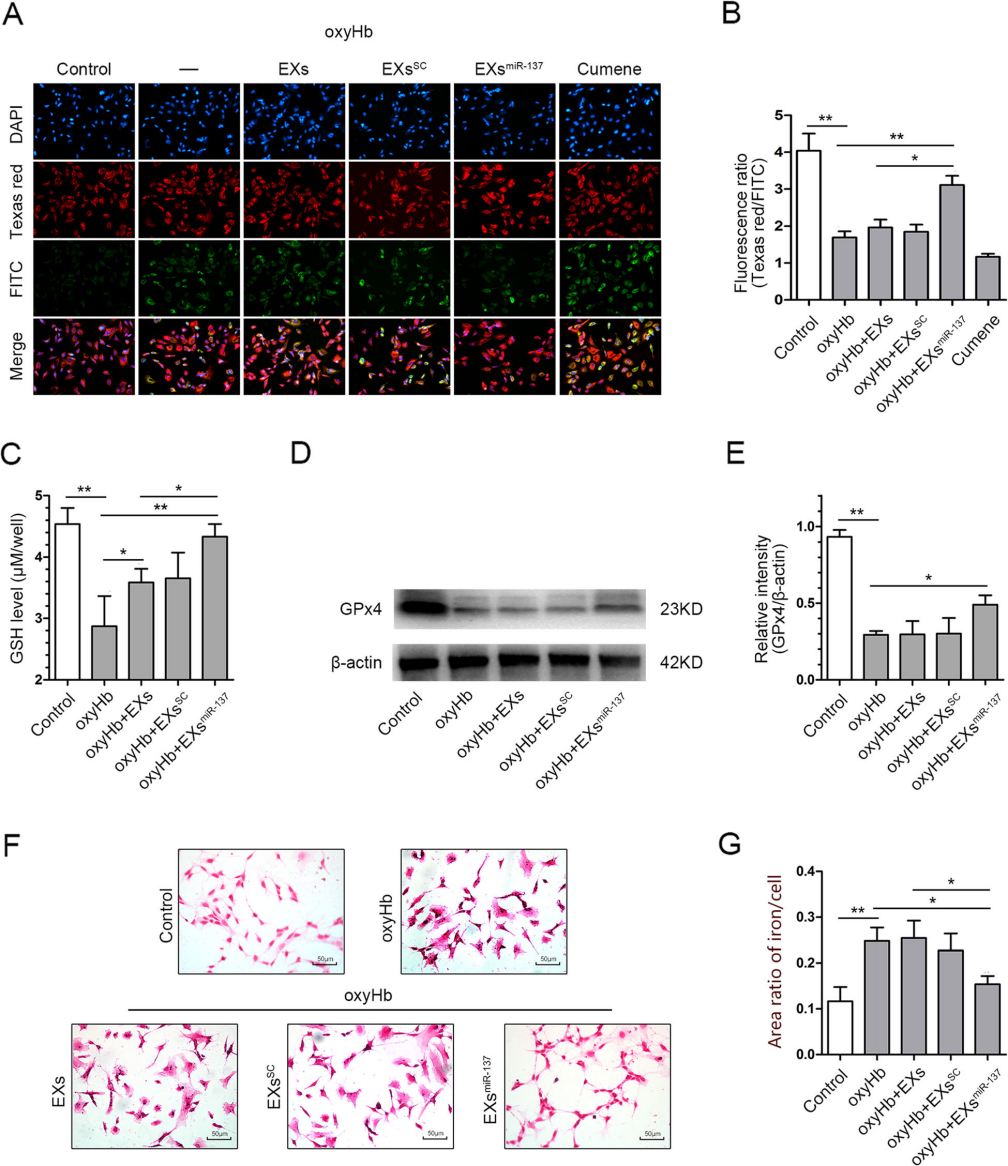
EPC-EXs^miR-137^ alleviated the oxyHb-induced ferroptosis in SH-SY5Y cells. **a** Representative micrographs of SH-SY5Y cells stained with the Lipid Peroxidation Kit and DAPI. **b** Quantitative analysis of the fluorescence ratio of Texas red/FITC which revealed the level of lipid peroxidation, *n* = 6. **c** Quantitative analysis of GSH content in the SH-SY5Y cells of each group, *n* = 6. **d** Representative bands of GPx4 and β-actin proteins detected by western blot. **e** Quantification of GPx4 expression in each group, β-actin served as the reference protein, *n* = 5. **f** Representative micrographs of iron staining in the SH-SY5Y cells. **g** Quantitative analysis of iron deposition in the cells from different groups, *n* = 4. *P < 0.01 and **P < 0.01

To determine the effects of EXs^miR-137^ on ferroptosis, we measured lipid peroxidation in oxyHb-treated SH-SY5Y cells. As shown in Fig. 6a and b, lipid peroxidation was increased after oxyHb treatment (*p* < 0.01 vs. control). EXs^miR-137^ decreased lipid peroxidation (*p* < 0.01 vs. oxyHb), while EXs and EXs^SC^ did not affect lipid peroxidation. The Cumene group served as the positive control group. The main antioxidants GSH and GPx4 were also measured. The GSH content in cells was decreased after oxyHb treatment (*p* < 0.01 vs. control). Administration of EXs and EXs^SC^ improved the GSH content (*p* < 0.05 vs. oxyHb), while EXs^miR-137^ were more effective than EXs (*p* < 0.05 vs. EXs, Fig. 6c). EXs^miR-137^ could also alleviate the reduction in the expression of GPx4 in the oxyHb group (*p* < 0.05 vs. oxyHb), while EXs and EXs^SC^ could not (Fig. 6d, e). Iron deposition, which can be used as another measure of ferroptosis, was increased in the oxyHb group (*p* < 0.01 vs. control). EXs^miR-137^ rather than EXs decreased the iron deposition in the oxyHb group (*p* < 0.05 vs. oxyHb, Fig. 6f, g). These results indicate that EXs^miR-137^ could inhibit oxyHb-induced ferroptosis in SH-SY5Y cells and that EXs had no effect.

### EPC-EXs^miR-137^ suppress the COX2/PGE2 pathway in oxyHb-treated SH-SY5Y cells

**Fig. 7:**
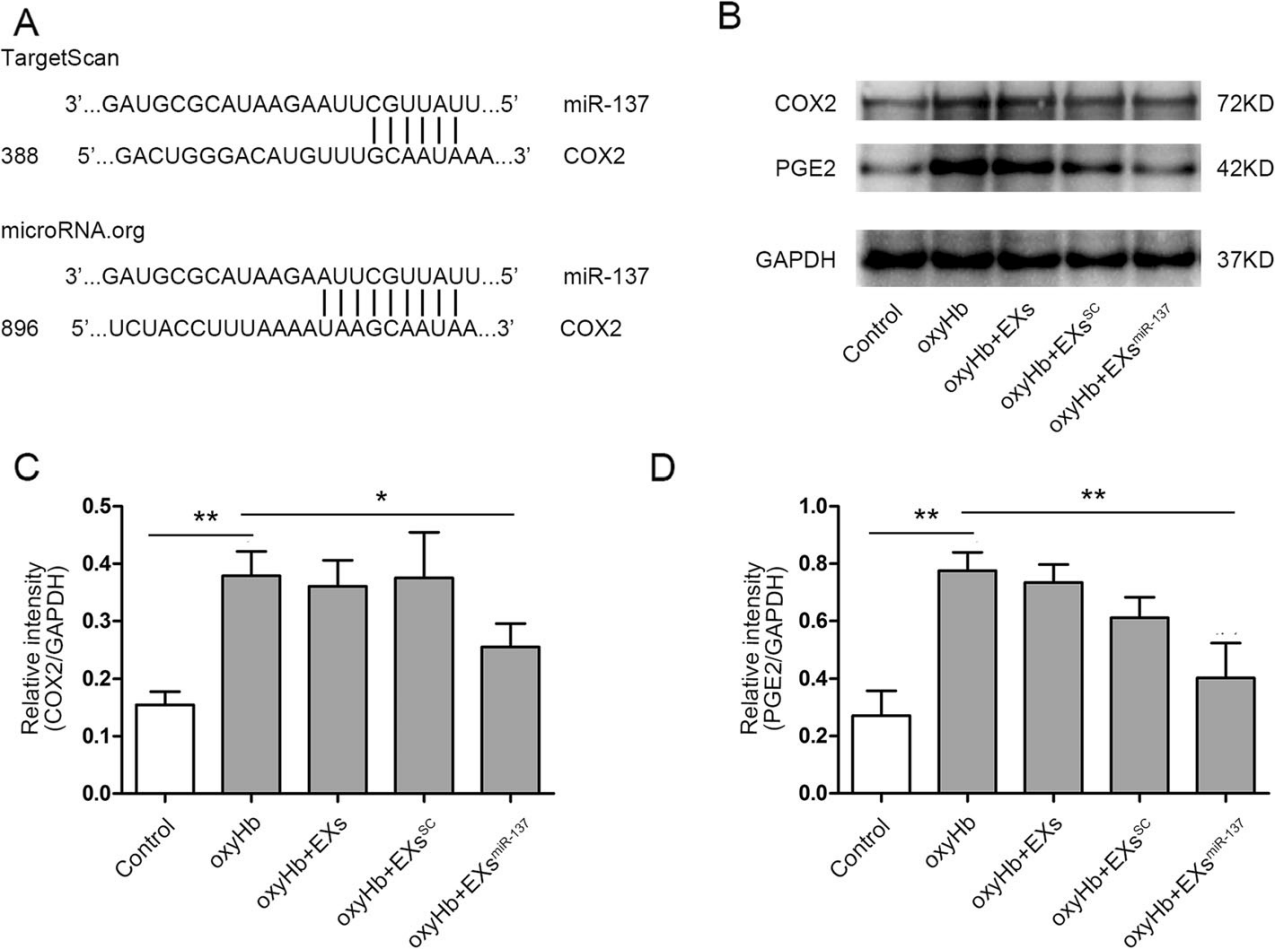
EPC-EXs^miR-137^ suppressed the activation of COX2/PGE2 pathway in oxyHb-treated SH-SY5Y cells. **a** The binding sites of miR-137 and COX2 predicted by TargetScan and microRNA.org. **b** Representative bands of COX2, PGE2, and β-actin measured by western blot, β-actin served as the reference protein. **c** Quantification of COX2 expression in each group, *n* = 4. **d** Quantification of PGE2 expression in each group, *n* = 4. *P < 0.01 and **P < 0.01

Using the bioinformatic databases TargetScan and microRNA.org, it was determined that miR-137 has binding sites in COX2, as shown in Fig. 7a. To explore if COX2 is affected by miR-137, the expression of COX2 and its product PGE2 were measured with western blot (Fig. 7b). The results show that both proteins were significantly increased in the oxyHb group (*p* < 0.01 vs. control). EXs^miR-137^ decreased the overexpression of COX2 (*p* < 0.05 vs. oxyHb) and PGE2 (*p* < 0.01 vs. oxyHb) induced by oxyHb treatment, while EXs and EXs^SC^ had no impact on the COX2/PGE2 pathway (Fig. 7c, d).

### Activation of the COX2/PGE2 pathway abolished the protective effect of EPC-EXs^miR-137^ against apoptosis and mitochondrial dysfunction in SH-SY5Y cells

**Fig. 8:**
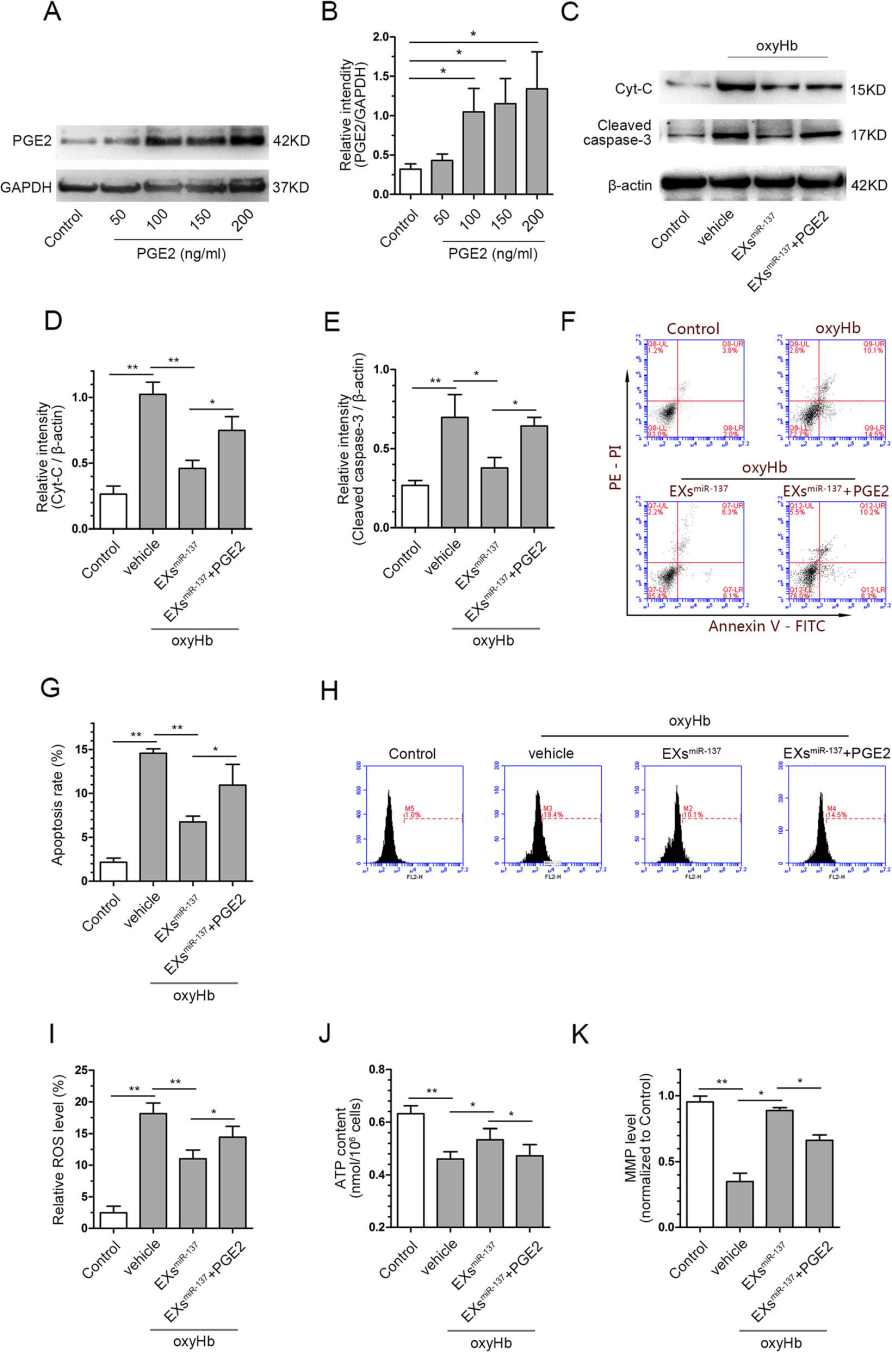
Activation of COX2/PGE2 pathway abolished the protection of EPC-EXs^miR-137^ against apoptosis and mitochondrial dysfunction. **a** Representative bands of PGE2 at different concentrations and GAPDH measured by western blot. **b** Quantification of PGE2 expression at different concentrations, the densities of the protein bands were normalized to GAPDH, *n* = 4. **c** Representative bands of Cyt-C, cleaved caspase-3, and β-actin proteins by western blot in each group, β-actin served as the reference protein. **d** Quantification of relative expression of Cyt-C in each group, *n* = 4. **e** Quantification of relative expression of cleaved caspase-3 in each group, *n* = 4. **f** Representative Annexin V/PE staining result measured by flow cytometry. **g** Summarized data of the apoptosis rate in each group, *n* = 4. **h** Representative DHE staining result measured by flow cytometry. **i** Summarized data of the relative ROS level in each group, *n* = 4. **j** Quantification of ATP content in the SH-SY5Y cells of each group, *n* = 6. **k** Quantification of MMP level in the SH-SY5Y cells of each group, *n* = 6. *P < 0.05 and **P < 0.01 vs. control group, *P < 0.01 and **P < 0.01

We explored if the activation of the COX2/PGE2 pathway could reverse the protective effects of EXs^miR-137^ using the recombinant protein PGE2. First, western blot showed that a concentration of 100 ng/ml could significantly increase the expression of PGE2 in SH-SY5Y cells (*p* < 0.05 vs. control, Fig. 8a, b). With regard to apoptosis, PGE2 could partially reverse the expression of Cyt-C and caspase-3 in the oxyHb+EXs^miR-137^ group (*p* < 0.05, Fig. 8c–e). The flow cytometry analysis also found that PGE2 increased the apoptosis rate of cells in the oxyHb+EXs^miR-137^ group (*p* < 0.05, Fig. 8f, g). With regard to mitochondrial function, the DHE staining showed that the ROS level in the oxyHb+EXs^miR-137^ group was increased after PGE2 administration (*p* < 0.05, Fig. 8h, i). The ATP content and MMP level in the SH-SY5Y cells in the oxyHb+EXs^miR-137^ group were also reduced after PGE2 administration (*p* < 0.05, Fig. 8j, k). These results indicate that the activation of the COX2/PGE2 pathway reversed the protective effects of EXs^miR-137^ against apoptosis and mitochondrial dysfunction in oxyHb-treated SH-SY5Y cells.

### Activation of the COX2/PGE2 pathway abolished the protective effect of EPC-EXs^miR-137^ against ferroptosis in SH-SY5Y cells

**Fig. 9:**
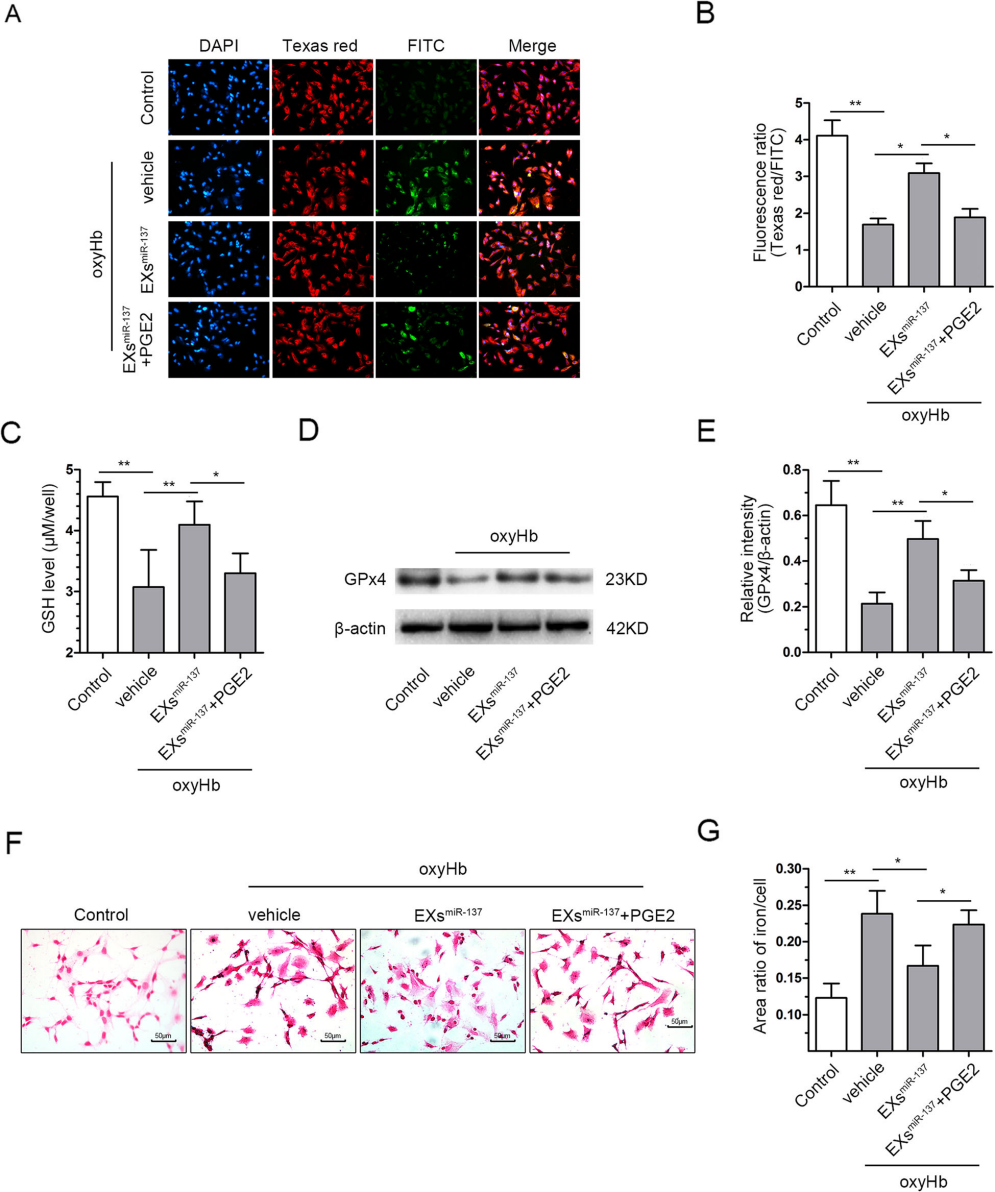
Activation of COX2/PGE2 pathway abolished the protection of EPC-EXs^miR-137^ against ferroptosis. **a** Representative micrographs of lipid peroxidation stain in SH-SY5Y cells of each group. **b** Quantitative analysis of the relative level of lipid peroxidation in each group, *n* = 6. **c** Quantitative analysis of GSH content in the SH-SY5Y cells of each group, *n* = 6. **d** Representative bands of GPx4 and β-actin proteins measured by western blot. **e** Quantification of GPx4 expression in each group, β-actin served as the reference protein, *n* = 5. **f** Representative micrographs of iron staining in the SH-SY5Y cells of each group. **g** Quantitative analysis of iron deposition in the cells from different groups, *n* = 4. *P < 0.01 and **P < 0.01

The results of the ferroptosis assay show that lipid peroxidation was partially exacerbated after PGE2 administration when compared with the oxyHb+EXs^miR-137^ group (*p* < 0.05, Fig. 9a, b). The GSH content and GPx4 were also decreased in the oxyHb+EXs^miR-137^+PGE2 group when compared with the oxyHb+EXs^miR-137^ group (*p* < 0.05, Fig. 9c–e). Iron deposition was partially increased in the oxyHb+EXs^miR-137^+PGE2 group compared to the oxyHb+EXs^miR-137^ group (*p* < 0.05, Fig. 9 f, g). These results indicate that the activation of the COX2/PGE2 pathway reversed the protective effects of EXs^miR-137^ against ferroptosis in oxyHb-treated neurons.

## Discussion

There are several novel findings in the present study: (1) EPC-EXs could be taken up by SH-SY5Y cells via caveolin-/clathrin-mediated pathways and macropinocytosis; (2) EPC-EXs alleviated oxyHb-induced apoptosis and mitochondrial dysfunction; (3) the miR-137 level was significantly decreased in oxyHb-treated SH-SY5Y cells; (4) EXs^miR-137^ improved the reduction in miR-137 levels induced by oxyHb; (5) miR-137 overexpression boost the protective effects of EPC-EXs in oxyHb-treated cells against apoptosis and mitochondrial dysfunction, and EXs^miR-137^ also inhibited oxyHb-induced ferroptosis; (6) EXs^miR-137^ suppressed the activation of the COX2/PGE2 pathway in oxyHb-treated cells; and (7) activation of the COX2/PGE2 pathway partially reversed the neuroprotective effect of EXs^miR-137^ in oxyHb-treated cells.

Our previous work showed that EPC-EXs had beneficial effects against H/R injury in endothelial cells by alleviating mitochondrial dysfunction [15]. However, there have been no studies of EPC-EXs in hemorrhagic stroke. EXs are small (30–150 nm) extracellular vesicles surrounded by phospholipid bilayers [24]. EXs contain various mRNAs, miRNAs, proteins, and other molecules. They can be secreted by nearly all types of cells and detected in almost all bodily fluids. In previous reports, EXs were found to be mainly taken up by living cells via clathrin- or caveolin-dependent endocytosis, macropinocytosis, phagocytosis, lipid raft-mediated internalization, and membrane fusion [25, 26]. In order to explore the effects of EPC-EXs on neurons in hemorrhagic stroke, we first determined the uptake mechanisms of EPC-EXs in SH-SY5Y cells with inhibitors of the pathways mentioned above. The results indicate that the caveolin- and clathrin-mediated pathways and macropinocytosis are involved in the uptake of EPC-EXs in SH-SY5Y cells. In addition, the uptake of EPC-EXs was comparable between normal and oxyHb-treated cells, meaning that oxyHb treatment did not influence EPC-EXs uptake.

The present work used oxyHb-treated SH-SY5Y cells to establish a model of hemorrhagic stroke in vitro and to explore the injury mechanisms of oxyHb as well as the potential effects of EPC-EXs. As a major component of blood, oxyHb is widely used in the study of hemorrhagic stroke, including ICH and SAH [5, 6]. The underlying mechanisms of oxyHb reported to cause secondary neurological damage in stroke include the removal of nitric oxide, activation of the Rho/Rho kinase pathway and protein kinase C, and the induction of ROS overproduction [27]. In addition, oxyHb was found to induce mitochondrial dysfunction in SAH, as in our previous report [4]. Hence, oxyHb serves as a key factor in the pathological mechanisms of hemorrhagic stroke and leads to various injuries to the central nervous system. In our study, oxyHb was verified to cause apoptosis in neurons, which was revealed by the number of apoptotic cells and expression of active caspase-3 and Cyt-C. OxyHb also induced mitochondrial dysfunction by decreasing the MMP level and ATP content and increasing ROS accumulation in cells. This was in accordance with several previous studies on SAH or ICH [28, 29]. In a study on ICH, researchers verified that oxyHb could cause neuronal ferroptosis, which is a novel iron-dependent form of non-apoptotic cell death [6, 30, 31]. In our study, ferroptosis was also induced by oxyHb in SH-SY5Y cells via obvious iron deposition, lipid peroxidation, and downregulation of antioxidant substances. These results indicate that oxyHb treatment might lead to neuronal injury, and alleviating oxyHb-induced injury might be beneficial for improving the outcome of hemorrhagic stroke.

As key mediators of intercellular communication, EXs participate in both normal physiological processes and the development of disease [32]. In studies of stroke, EXs secreted by MSCs are reported to represent a major mechanism by which MSCs improve neurovascular remodeling and neurological function after ischemic stroke [33]. Astrocyte-derived EXs have been shown to participate in the regulation of neuronal apoptosis and autophagy in ischemic stroke [34]. In the present study, we determined the effects of EPC-EXs on SH-SY5Y cells after oxyHb treatment. First, the EPC-EXs were found to relieve apoptosis and mitochondrial dysfunction in oxyHb-treated cells, which was in agreement with the conclusion of our previous study on H/R [15]. Interestingly, EXs partially restored the GSH level, an important antioxidant in living cells, in oxyHb-treated cells; however, they could not improve the iron deposition and lipid peroxidation, which are the two major characteristics of ferroptosis. So, EPC-EXs were beneficial for the alleviation of oxyHb-induced apoptosis and mitochondrial damage in neurons but were not sufficient to suppress the occurrence of ferroptosis.

In a study of melanoma, miR-137 was found to play a novel and indispensable role in ferroptosis [35]. Moreover, EXs have been used as immunotherapy and potential biomarkers for cancer and neurodegenerative diseases and are also involved in the delivery of pharmacological agents or genes [36, 37]. For instance, the EXs derived from miR-133b-overexpressing MSCs exhibited anti-apoptotic effects in rats after ICH [38]. Thus, we suspected that miR-137 overexpression might enrich the protective effects of EPC-EXs because of its critical role in the development, differentiation, maturation, and pathological processes of the nervous system [17]. In studies of ischemic stroke and spinal cord injury, miR-137 was reported to benefit the repair of the nervous system via various downstream targets [18, 39]. In the present work, we first found that miR-137 was downregulated in oxyHb-treated SH-SY5Y cells and that transfection with EPC-EXs did not change the level of miR-137 in the cells, suggesting that miR-137 was involved in oxyHb-induced neuronal injuries and that the effects of EXs were independent on miR-137. The miR-137-overexpressing EXs were formed by lentiviral transfection. Interestingly, we found that EXs^miR-137^ performed better than EXs alone at inhibiting oxyHb-induced apoptosis and mitochondrial dysfunction. This indicates that the combination of miR-137 and EPC-EXs would generate better protection against oxyHb-induced injury in SH-SY5Y cells. In addition, we found that the same number of EPC-EXs, EPC-EXs^SC^, and EPC-EXs^miR-210^ were taken up by oxyHb-injured cells, which further supported the hypothesis that miR-137 plays a role in the beneficial effects of EPC-EXs rather than enhancing the uptake of EXs. We next focused on the effect of EXs^miR-137^ on ferroptosis. Surprisingly, EXs^miR-137^ was found to reduce lipid peroxidation and iron deposition in oxyHb-treated cells, while EXs alone did not exhibit this protective effect. Moreover, EXs^miR-137^ further increased the GSH content and GPx4 expression in SH-SY5Y cells following oxyHb treatment when compared with EXs. The increase in GSH and GPx4 levels undoubtedly contributed to the alleviation of lipid peroxidation. With respect to the regulation of GSH and GPx4, the inhibition of glutaminolysis by miR-137 might serve as one mechanism since studies have demonstrated that inhibiting glutaminolysis could restrain ferroptosis by decreasing the formation of lipid oxidized membrane lipids [30, 40]. Another possibility would be that GPx4, which is the major anti-lipid oxidation enzyme in ferroptosis and is mainly synthesized with GSH [41], was upregulated by EXs^miR-137^, and that GSH was compensatorily upregulated. However, these findings suggest that miR-137 can alleviate ferroptosis and boost the protective effects of EPCs-EXs against apoptosis as well as mitochondrial damage in neurons after oxyHb treatment, and this strengthens the viewpoint that one single miRNA could simultaneously regulate several programmed cell death mechanisms.

Since miRNAs can regulate multiple pathways, it was of interest to explore the putative targets involved in miR-137-mediated neuroprotection. COX2, which belongs to the cyclooxygenase family, was verified as a direct target of miR-137 in tumor inhibition of glioblastoma carcinogenesis [42]. Using bioinformatic methods, we confirmed that COX2 was a potential target of miR-137. COX-2 has been reported to be constitutively upregulated in several pathological processes, such as inflammation, tumorigenesis, and other injuries [43]. In ICH, COX2 was found to be induced in the ipsilateral hemisphere of rats at 6 h after ICH onset; however, its expression was not verified to be a major regulator of blood flow or edema formation [44]. In our in vitro model, COX2 and its main product PGE2 were overexpressed in SH-SY5Y cells following oxyHb treatment, which indicates that the COX2/PGE2 pathway participates in the pathological mechanisms of oxyHb in neurons. In a study of gastric carcinogenesis, COX2 was reported to be suppressed by miR-137, which mediated the tumor-suppressive effects [20]. In addition, inhibition of COX2 has been shown to alleviate brain injury in SAH or oxygen/glucose deprivation and reperfusion models [45, 46]. Thus, we hypothesized that miR-137 might also activate the COX2/PGE2 pathway to affect the outcomes after oxyHb treatment. Indeed, EXs^miR-137^ decreased the overexpression of both COX2 and PGE2 proteins in oxyHb-treated cells, while EXs did not change their expression level. Moreover, activation of the COX2/PGE2 pathway with recombinational PGE2 protein partially reversed the neuroprotective effects of EXs^miR-137^ on apoptosis and mitochondrial dysfunction, which further confirmed our hypothesis. In studies of ICH, COX2 and PGE2 were both reported to be involved in ferroptosis in the brain. Qian et al. considered that COX2 could serve as a biomarker of ferroptosis [6, 47]. Thus, we considered that the COX2/PGE2 pathway might also participate in the regulation of the mechanism of miR-137 in ferroptosis. In oxyHb-treated cells, activation of the COX2/PGE2 pathway reversed the reduction in iron accumulation and lipid peroxidation after EXs^miR-137^ co-incubation, and GSH and GPx4 levels were also decreased, suggesting that the inhibition of ferroptosis by EXs^miR-137^ could be reversed by activation of the COX2/PGE2 pathway. Taken together, the neuroprotective effect of EXs^miR-137^ against apoptosis, mitochondrial dysfunction, and ferroptosis in oxyHb-treated SH-SY5Y cells occurs partially via the miR-137-COX2/PGE2 pathway.

There are some limitations in the present work: (1) Although the SH-SY5Y cells were widely used in studies on neuronal injuries [48, 49], the cells express the markers of both neuronal and glial cell. So primary neurons would be selected in the following work to eliminate the potential involvement of glial cells. (2) Our hypothesis was only tested in a cell line in vitro, and thus, further tests should be carried out in vivo in the future.

## Conclusions

The present work showed that the caveolin- and clathrin-mediated pathways and macropinocytosis are involved in the uptake of EPC-EXs in SH-SY5Y cells. In addition, miR-137-overexpression was for the first time found to boost the neuroprotective effects of EPC-EXs on oxyHb-induced miR-137 downregulation, apoptosis, and mitochondrial dysfunction. Additionally, EXs^miR-137^ could suppress the oxyHb-induced ferroptosis in SH-SY5Y cells while EXs alone could not. We also found that the protective effects of EXs^miR-137^ were partially dependent on the miR-137–COX2/PGE2 pathway.

## Data Availability

The datasets used and/or analyzed during the current study are available from the corresponding author on reasonable request.
